# Failure of Working Memory Training to Enhance Cognition or Intelligence

**DOI:** 10.1371/journal.pone.0063614

**Published:** 2013-05-22

**Authors:** Todd W. Thompson, Michael L. Waskom, Keri-Lee A. Garel, Carlos Cardenas-Iniguez, Gretchen O. Reynolds, Rebecca Winter, Patricia Chang, Kiersten Pollard, Nupur Lala, George A. Alvarez, John D. E. Gabrieli

**Affiliations:** 1 Department of Brain and Cognitive Sciences, Massachusetts Institute of Technology, Cambridge, Massachusetts, United States of America; 2 McGovern Institute for Brain Research, Massachusetts Institute of Technology, Cambridge, Massachusetts, United States of America; 3 Department of Psychology and Center for Mind, Brain, and Computation, Stanford University, Stanford, California, United States of America; 4 Department of Psychology, University of Chicago, Chicago, Illinois, United States of America; 5 Department of Psychology, Boston University, Boston, Massachusetts, United States of America; 6 Department of Psychiatry, Mount Sinai School of Medicine, New York City, New York, United States of America; 7 Department of Pediatrics Research, Children’s Cancer Hospital, MD Anderson Cancer Center, Houston, Texas, United States of America; 8 Department of Psychology, Harvard University, Cambridge, Massachusetts, United States of America; University of Leuven, Belgium

## Abstract

Fluid intelligence is important for successful functioning in the modern world, but much evidence suggests that fluid intelligence is largely immutable after childhood. Recently, however, researchers have reported gains in fluid intelligence after multiple sessions of adaptive working memory training in adults. The current study attempted to replicate and expand those results by administering a broad assessment of cognitive abilities and personality traits to young adults who underwent 20 sessions of an adaptive dual n-back working memory training program and comparing their post-training performance on those tests to a matched set of young adults who underwent 20 sessions of an adaptive attentional tracking program. Pre- and post-training measurements of fluid intelligence, standardized intelligence tests, speed of processing, reading skills, and other tests of working memory were assessed. Both training groups exhibited substantial and specific improvements on the trained tasks that persisted for at least 6 months post-training, but no transfer of improvement was observed to any of the non-trained measurements when compared to a third untrained group serving as a passive control. These findings fail to support the idea that adaptive working memory training in healthy young adults enhances working memory capacity in non-trained tasks, fluid intelligence, or other measures of cognitive abilities.

## Introduction

A fundamental question of both theoretical and practical interest is whether the basic human cognitive abilities that underlie many aspects of learning, memory, thinking, and performance can be enhanced in adults. It has long been thought that the combination of genetics and early environment substantially determines life-long individual differences in generalizable cognitive abilities (i.e., abilities that support and limit performance on a wide range of tasks). Because standardized intelligence quotient (IQ) scores predict performance on a wide range of cognitive tasks and educational achievements [1], IQ scores are often used as an index of general cognitive abilities. Such IQ measures exhibit substantial correlations from late childhood through adulthood (e.g., IQ scores were estimated to correlate 0.73 from ages 11 through 77 in a longitudinal study [2]). These observations suggest that variation in general cognitive abilities is determined, to a large extent, by late childhood or early adolescence. This fixedness of cognitive ability has seemed especially strong for fluid intelligence (the ability to solve novel problems), relative to crystallized intelligence (the ability to apply specific knowledge, skills, and experience). In part this is because scores on tests of crystallized intelligence can be improved by, for example, instructing a student on the vocabulary that the crystallized intelligence tests typically evaluate, but also in part because fluid intelligence has typically been considered as more biologically determined than crystallized intelligence [3], [4].

More recently, evidence has emerged indicating some plasticity in IQ and its neural bases. One study reported that verbal and performance IQ scores, as well as their neural correlates, exhibited some fluctuation across the teenage years, rather than remaining static [5]. A particularly influential study by Jaeggi and colleagues not only reported plasticity in adult fluid intelligence, but also defined a specific cognitive training program that enhanced fluid intelligence [6]. In this study, young adults performed a working memory (WM) task for about 25 minutes per day for up to 19 days. The WM task trained WM capacity, defined here as the amount of goal-relevant information that could be simultaneously maintained and processed. Specifically, the training task used a “dual n-back” paradigm in which participants simultaneously heard letters and saw spatial locations presented one after another. Their task was to respond whenever a presented stimulus was identical to the stimulus presented *n* trials ago (e.g., in a dual 2-back, subjects responded whenever the current spatial position *or* the current auditory stimulus matched the presentation from 2 trials earlier). Performance improved on the trained WM task, and most importantly, there were significant post-training gains on a measure of fluid intelligence. Thus, the learned skill in performing the WM task *transferred* to a growth in fluid intelligence. These findings were exciting because they offered a way to enhance adult fluid intelligence, previously viewed as static. Because superior fluid intelligence is associated with superior performance on many cognitive and learning measures, these findings suggested a practical way by which cognitive training might lead to widespread gains in cognitive ability.

Two aspects of the WM training that yielded a gain in fluid intelligence seem important. First, it trained a cognitive construct (working memory) that has been associated with fluid intelligence in many studies [7], [8], such that transfer might be expected. Generally, transfer might be expected from one task to another when those two tasks share common cognitive mechanisms, either through reliance on similar cognitive processes, or through a shared neural substrate. Among adults, greater WM capacity is associated with superior performance in a broad range of high-level cognitive domains, including reading comprehension, problem solving, and inhibitory control [9] and so is thought to reflect central executive capability [7]. Thus, it is plausible that WM training might improve central executive capability and/or fluid intelligence. Second, the WM training was adaptive, such that the span (or the number of intervening stimuli) increased between the presented target and its potential match as a participant performed better on the task, or decreased as the participant performed worse on the task. Such an adaptive design makes certain that the participant constantly performs at a challenging but not frustrating level. These types of adaptive designs have been a core feature of effective WM training (reviewed in [10]). Indeed, this adaptive design resulted in more than a doubling of WM capacity on the trained WM task [6]. Thus, the training program that raised fluid intelligence was theoretically motivated and effective in design.

The provocative finding that a WM capacity training task can increase fluid IQ in adults raised several questions [11]. First, the control group was a no-contact group that was tested on the fluid IQ measure with a comparable testing interval. The lack of an active training regime for the control group leaves open questions of specificity (e.g., would any demanding training program yield such a gain in fluid IQ? are there correlated factors such as motivation associated with the training experience that influence transfer?). Second, transfer was only demonstrated on one specific test of fluid IQ, leaving open the question of the scope and limits of the transfer of cognitive gains from the WM training program (e.g., would such transfer occur for another measure of fluid IQ? would it occur for measures of crystallized IQ or other cognitive abilities such as processing speed?). Third, does such WM training result in enduring gains that are sustained well after the training program, or must the training be continued to maintain gains on either WM or fluid intelligence measures?

After publication of the Jaeggi et al. study [6], several subsequent studies have examined the influence of WM training on fluid IQ and other types of cognition. One study, using a similarly adaptive WM training program, reported no gains on fluid IQ, but did report gains in reading and cognitive control [12]. Two other studies, using dual n-back training tasks identical to Jaeggi et al. [6] failed to find any gains on fluid IQ [13], [14]. Other research was more consistent with the original findings, including (1) a partial replication in children, in which participants who exhibited gains on the WM training task also exhibited gains on a fluid IQ measure [15]; (2) a report of both fluid intelligence improvements and corresponding changes in EEG measures after WM training which included the dual n-back among other tasks [16]; and (3) a finding of transfer from both single n-back and dual n-back training to fluid intelligence gains, but with effects mediated by conscientiousness and neuroticism personality factors ([17], originally reported in [18]).

Because the transfer from WM training to fluid intelligence is both controversial and important, we aimed to replicate and extend the finding that WM training enhances fluid IQ. Two groups of young adults, stratified so as to be equated on initial fluid IQ scores, were randomly assigned to two conditions (a randomized controlled trial or RCT). The experimental group performed the dual n-back task (as in the original Jaeggi et al., 2008 study [6]) for approximately 40 minutes per day, 5 days per week for 4 weeks (20 sessions of 30 blocks per session, exceeding the maximum of 19 sessions of 20 blocks per day in the original Jaeggi et al., 2008 study). An active control group performed a visuospatial skill learning task, multiple object tracking (or MOT), on an identical training schedule. We also tested a no-contact group equated for initial fluid IQ in case both kinds of training enhanced cognitive abilities.

Tests of cognition were administered before and after training (or after an equal duration of time for the no-contact group) in order to evaluate the benefits of the training. Two tests were versions of the training tasks (dual n-back and MOT). We hypothesized that, as in prior studies, there would be significant improvements on the trained tasks, and that because the tasks were quite different, there would be selective gains on the trained relative to the untrained tasks for both groups. We also asked in a subset of participants whether the skills gained during training would endure over a 6-month period without further training.

A second set of tests measured *near transfer*, gains on untrained WM capacity measures that were conceptually similar to the dual n-back training task. In Baddeley and Hitch’s original model of working memory [19], working memory has separate and independent slave subsystems (the *phonological loop* and *visuospatial sketchpad*), and these modality-specific storage systems are coordinated by a modality-independent *central executive*. Evidence for transfer from trained WM tasks to non-trained WM tasks suggests that these WM tasks share underlying processes (e.g., [20]–[22]). In the present study, we selected two widely studied tasks, Operation Span and Reading Span [23], which are similar to the dual n-back task because all three tasks measure complex working memory (CWM). All three of these CWM tasks involve encoding a presented stimulus, performing some sort of updating/manipulation (validating a math problem, assessing the sensibility of a sentence, or updating the numerical position of the rehearsed stimuli), and retrieval (either of all the encoded stimuli in the case of the span tasks, or of the nth-back stimuli in the dual n-back task). Transfer of any broad gain in WM capacity would be expected on the Operation Span and Reading Span tasks if dual n-back training enhances either the capacities of either the phonological loop (responsible for the storage of verbally encoded material for subsequent retrieval) or of the central executive (responsible for the updating and manipulation components of the tasks).

The Operation and Reading Span tasks were selected specifically because there is considerable evidence that these tasks measure the central executive component of WM. Performance on these tasks has been correlated with performance on a broad range of other tasks, including tests of verbal, numerical and spatial reasoning, matrix reasoning such as the Raven’s Progressive Matrices, processing speed, and general knowledge [24], [25]. Observing an improvement on these CWM measures following dual n-back training could lend support to the idea that dual n-back training increases CWM capacity.

In addition to the assessment of trained tasks and the near-transfer tasks, a third set of tests measured *far transfer*, gains on measures that were dissimilar to the WM training task, including measures of fluid IQ, crystallized IQ, reading skill, and processing speed. Although the common components between the dual n-back task and the far-transfer tasks are not as apparent as those in the near-transfer tasks, there are often strong correlations between measures of CWM and fluid intelligence, which suggests that there are shared mental processes [8], [9], [26]. The prior report that training on the dual n-back task enhanced scores on matrix reasoning tasks further supports the idea that CWM capacity and fluid intelligence share underlying processes [6]. Additional measures of far transfer were selected to determine the scope and limits of transfer from WM training, as well as a specific report that similar training enhanced reading skills [12].

We also examined the possibility of individual personality differences among participants modulating either training or transfer, in an attempt to illuminate the reasons behind the mixed results so far reported in the WM training literature. Greater conscientiousness has been reported to predict greater improvement on a dual n-back task during training, but lesser transfer of training to a measure of fluid intelligence transfer [17]. We therefore measured conscientiousness in all participants as the “Conscientiousness” factor from the Big Five personality test [27]. We also examined two additional characteristics of all participants. We measured implicit theories of intelligence, defined as the extent to which a person believes that intelligence is a fixed or innate trait, as opposed to viewing intelligence as a capacity that can incrementally grow through effort and learning. Those who view intelligence as improvable with effort are said to have a “growth mindset” [28]. We also measured “grit”, defined as perseverance and passion for long-term goals [29]. Both growth mindset [30] and greater grit [29] have been associated with better performance and learning in a variety of settings.

## Methods

### Participants, Recruitment, and Group Assignment

Participants were recruited through web advertisements, physical flyers, and e-mail to the Northeastern and Tufts college mailing lists. Participants were required to be adults between the ages of 18 and 45, right-handed, in good health, and not taking any drugs. All participants provided informed, written consent before participation. This study was approved by the Massachusetts Institute of Technology Institutional Review Board (PI: Leigh Firn).

After recruiting each participant, we performed pre-training behavioral testing and determined his or her group assignment (Table 1). Each incoming participant was paired with another participant based on age, gender, and score on the Raven’s Advanced Progressive Matrices (RAPM) task, and each member of that pair was randomly assigned to either the n-back or the MOT training group. The No-Contact group was recruited separately, but in the same fashion, and matched to a training pair by gender and initial RAPM. Because of this matching procedure, the No-Contract group was slightly, but significantly, older than the two training groups (Table 1). The No-Contact group averaged 1.8 years older than the other two groups [F(2,55) = 3.37, p<.05]. However, the three groups did not differ significantly by gender or RAPM scores [F(2,55) <1, p>.8], nor did they differ on the full IQ score from the Wechsler Abbreviated Scale of Intelligence [31], administered as part of the pre-training battery [F(2,55) <1, p>.4].

**Table 1:** Participant Characteristics.

TrainingGroup	AverageAge	Gender	RAPM (SD)	Full-4 IQ (SD)
Dual n-back	21.2	7 M, 13 F	13.3 (2.1)	120.8 (10.8)
Multiple Object Tracking	21.3	8 M, 11 F	13.6 (2.0)	120.7 (7.0)
No Contact	23.1	7 M, 12 F	13.3 (2.2)	117.6 (7.4)

Participants were assigned to treatment groups based primarily on gender and initial score (out of 17) on the Ravens Advanced Progressive Matrices problems (RAPM).

Eighteen potential participants either dropped out of the study or were excluded after initial testing was completed. Two participants assigned to the dual n-back condition voluntarily withdrew (one after 5 days of training, the other after 9 days); no other participants had begun training when they were excluded or withdrew. Five participants provided initial behavioral data during the process of collecting the passive-control group, but were not included because they were not well-matched to an unmatched member of the other two groups based on Ravens score. The remaining eleven subjects were not included for a variety of logistical reasons, including difficulties aligning schedules with the experimenters, claustrophobia or excessive movement in fMRI scanning sessions, or repeatedly skipping appointments. Although we attempted to perform all behavioral measures with all included participants, in a few cases there were technical problems in administering some measures to some participants (these are noted in Tables 2 and 3).

**Table 2:** Initial Task Correlations with Training Tasks.

Behavioral Task	Correlation with Initial Dualn-Back d’	p-value	Correlation with InitialMOT Speed	p-value
Initial MOT Speed^a^	0.19	.149	N/A	N/A
*Complex Working Memory Measures*				
Operation Span Score^a^	0.36	**0.006**	0.26	0.055
Reading Span Score	0.27	**0.043**	0.14	0.312
Combined Span Score^a^	0.36	**0.006**	0.22	0.100
*Fluid Intelligence Measures*				
RAPM Score (out of 17)	0.50	**<.001**	0.19	0.160
*Weschler Abbreviated Scale of Intelligence Subtests*				
WASI Blocks	0.42	**<.001**	0.41	**0.001**
WASI Matrices	0.28	**0.033**	0.10	0.479
WASI Similarities	0.23	0.086	0.08	0.540
WASI Vocabulary	0.24	0.073	0.16	0.222
*Reading Measures*				
Nelson Denny Reading Rate	0.16	0.241	0.13	0.337
Nelson Denny Comprehension	0.28	**0.031**	0.24	0.069
*Speed of Processing Tasks*				
Woodcock Johnson III Pair Cancellation	0.29	**0.029**	0.28	**0.033**
Woodcock Johnson III Visual Matching	0.18	0.170	0.46	**<.001**
Digit/Symbol Coding	0.21	0.112	0.23	0.081
*Personality Measurements*				
Conscientiousness^a^	−0.03	0.818	0.01	0.938
Dweck	−0.10	0.438	−0.03	0.829
Grit	0.117	0.384	0.04	0.795

Correlations between initial scores on the two training tasks and the behavioral outcome measures are shown. Statistically significant (uncorrected for multiple comparisons) correlations are bolded. Unless otherwise specified, correlations are across 58 participants (19 passive control, 19 multiple object tracking, 20 dual n-back).

a –19 dual n-back measurements.

**Table 3:** Transfer from Trained Tasks.

Task	n-back pre-test score (SEM)	n-back post-test score (SEM)	MOT pre-test score (SEM)	MOT post-test score (SEM)	Control pre-test score (SEM)	Control post-test score (SEM)	n-back/MOT training interaction p-value	3-way interactionp-value	Minimum Detectable Effect Size (sensitivity)
*Trained Tasks*									
**d’ (2–6 back)**	**1.09 (.09)**	**2.60 (.16)**	**1.14 (.12)**	**1.38 (.15)**	**1.15 (.11)**	**1.41 (.14)**	**<.0001**	**<.0001**	**.19**
**MOT Speed** a	**6.28 (.63)**	**7.19 (.85)**	**6.76 (.68)**	**14.3 (1.31)**	**6.13 (1.0)**	**6.36 (.96)**	**<.0001**	**<.0001**	**.08**
*Complex Working Memory Measures (Near Transfer)*									
Operation Span^b^	47.4 (3.15)	58.5 (2.52)	47 (3.06)	51.7 (2.7)	51.6 (4.0)	60.1 (3.28)	.210	.384	.19
Reading Span^c^	48 (3.06)	52.4 (2.68)	41.8 (3.4)	39.3 (3.38)	46.1 (4.10)	47.4 (4.48)	.176	.306	.11
Combined Span^b^	95.9 (5.69)	110.8 (4.66)	88.8 (6.06)	91 (5.36)	97.8 (6.64)	107.5 (7.15)	.154	.267	.13
*Fluid Intelligence Tasks (Far Transfer)*									
RAPM	13.3 (0.47)	13.2 (0.67)	13.6 (0.46)	13.3 (0.5)	13.3 (0.49)	12.7 (0.62)	.827	.861	.29
*WASI/WAIS Subtasks (Far Transfer)*									
Vocabulary	14.5 (0.48)	16.1 (0.6)	14.3 (0.33)	15.9 (0.45)	13.7 (0.44)	15.4 (0.34)	.871	.983	.27
Blocks	13 (0.35)	13.9 (0.57)	13.3 (0.45)	14.6 (0.59)	12.2 (0.52)	13.3 (0.61)	.464	.737	.16
Similarities	13 (0.45)	14.2 (0.49)	13 (0.4)	13.8 (0.51)	13.3 (0.25)	14.1 (0.46)	.558	.748	.29
**Matrix Reasoning**	**13 (0.4)**	**13.4 (0.6)**	**12.9 (0.37)**	**14.7 (0.49)**	**12.6 (0.45)**	**13.7 (0.45)**	**.024**	**.096**	**.32**
*Nelson-Denny Reading Measurements (Far Transfer)*									
Comprehension*^d^*	236 (2.81)	236 (2.58)	237 (2.25)	237 (2.62)	240 (1.88)	240 (1.97)	.980	.979	.32
Reading Rate*^d^*	209 (4.1)	216 (4.53)	213 (5.82)	221 (5.74)	214 (5.08)	216 (4.49)	.684	.447	.19
*Speed of Processing Tasks (Far Transfer)*									
Digit Symbol Coding	11.8 (0.59)	13.3 (0.64)	10.9 (0.64)	11.9 (0.65)	11.4 (0.51)	12.6 (0.48)	.421	.694	.20
Visual Matching*^d^*	105 (2.2)	109 (1.95)	106 (3.75)	110 (2.81)	105 (3.17)	106 (3.44)	.851	.273	.14
Pair Cancellation*^d^*	98.8 (2.34)	105 (2.47)	97.6 (2.77)	102 (2.66)	97.7 (2.44)	104 (2.29)	.671	.797	.29

Pre- and post-testing means and standard errors of the means are presented for each treatment group. The interaction terms from repeated-measures ANOVAs show significant differences between treatments. Statistically significant (uncorrected for multiple comparisons) results are bolded. Unless otherwise specified, analyses include 19 passive control participants, 19 MOT participants, and 20 dual n-back participants.

a -19 dual n-back.

b -14 passive control, 19 dual n-back.

c –14 passive control.

d – 18 passive control.

### Participant Payment

Participants in the training groups were paid $20 per training session, with a $20 bonus per week for completing all five training sessions in that week. All participants were paid $20 per hour for behavioral testing, and $30 per hour for imaging sessions (data from imaging sessions are reported separately).

### Overall Experiment Design

After recruitment, participants underwent approximately six hours of behavioral testing spread across three days and two hours of structural and functional magnetic resonance imaging. If a participant was assigned to one of the two active training conditions, they then completed twenty sessions of adaptive training on campus.

After training was completed, post-training behavioral testing and imaging were administered as soon as possible. (Average number of days between last training session and post-training testing was 4.3 days, with a minimum of 0 days and maximum of 14 days. Two participants were tested on the final training day, with at least 3 hours between the last training session and the post-testing session; all other participants were tested at least a day after the last training session. This time was not significantly different between groups [t(37) = .2, p>.8]). Participants in the active training conditions were asked to return approximately six months after the completion of training later to examine the status of their improvement on the trained tasks. (Average number of days before the follow-up testing was 187 days, with a minimum of 122 days and maximum of 252 days. This time was not significantly different between groups [t(19) = .78, p>.4]). Although some participants in each training group were unable to return for follow-up testing (primarily due to post-graduation dispersal), data from 11 participants in the MOT training group and 10 participants in the n-back training group were collected. For behavioral measurements in which the participant’s score was evaluated by the tester (e.g., the vocabulary sections of the WASI), testers were blinded to the participant’s training condition.

### Behavioral Testing - Trained Tasks

To establish baseline measures of the two possible training tasks and test for transfer or practice effects from one training condition to another, performance on both training tasks was evaluated before and after the training period.

#### Baseline dual n-back

Implementation of the adaptive dual n-back training task followed Jaeggi et al., 2008 [6]. An auditory letter and a visual square were simultaneously presented for 500 ms, followed by a 2500 ms response period. Letters were chosen from the consonants B, F, H, J, M, Q, R, and W to maximize auditory discriminability between letters. Squares were presented at one of eight positions evenly spaced around the periphery of the screen. Participants responded when one or both of the current stimuli matched a stimulus presented *n* trials ago. Auditory matches were identified with the index finger of the right hand, and visual matches were identified with the middle finger of the right hand. No response was required on trials that did not match the target, and either response could be made on trials where both stimuli matched. Each block presented *n +*20 trials, containing four auditory target trials, four visual target trials, and two trials where both auditory and visual stimuli matched. For baseline testing of the dual n-back task, participants completed 30 blocks of dual n-back trials, with 5 blocks of each level from 1-Back to 6-Back presented in a counter-balanced pseudorandom order. Participants were allowed to take breaks between blocks as needed. In order to control for response biases between subjects, a sensitivity index (d’) was calculated at each level from 1-back to 6-back for each participant [32]. Because participants in all three groups scored highly on the first level of the n-back without any practice, the dependent measure used to evaluate improvement was calculated by averaging the d’ scores from 2-back to 6-back for each participant.

#### Multiple object tracking

To assess the maximum speed at which participants could reliably track moving objects, we followed the general techniques from Alvarez & Franconeri, 2007 [33]. Participants were asked to track 4 dots among 12 distractor dots. At the beginning of each trial, 4 target dots were identified in green for 500 ms while all dots remained stationary. For the next 2500 ms, all 16 dots moved while the target dots remained identified in green. At that point, the 4 target dots turned black, and for the remaining 8500 ms of the trial, the target dots appeared identical to the distractor dots while the participant attempted to remember which dots were targets. Finally, participants identified the 4 tracked dots using a mouse and were given feedback.

The initial speed at which items moved for each participant was determined by a self-assessment task in which participants used the cursor keys to make targets move slower or faster and reported the speed at which they thought they could reliably track four targets. This was followed by a thresholding procedure over the following 90 trials in which the speed of the moving dots increased by.5 degrees of visual angle/second every time two trials in a row were answered correctly, and decreased by.5 degrees/second every time two trials in a row were answered incorrectly. To count as a correct trial, all 4 targets were required to be identified correctly. Participants were allowed to take breaks, as needed. The speed of the final trial was the dependent variable.

### Near Transfer Tasks - Working Memory Capacity

#### Automated operation span (complex WM capacity) [34]

Participants were presented with alternating letters and math equations, and asked to remember the letters while assessing whether each math equation was valid. Set sizes ranged from 3-letters to 7-letters, with each set size presented for 3 trials over the course of the task, in a random order. At the end of each trial, participants reported the letters in the order they were presented. The dependent measure was the “score” variable reported from the ePrime program, which is the sum of all perfectly remembered letter sets. One dual n-back participant’s pre-training Operation Span score was excluded for falling more than 3 standard deviations below the group average, whereas all of this participant’s other behavioral measurements were near the group average, including the cognitively similar Reading Span score. It is unclear whether this score represented some sort of experimenter error in data collection or participant confusion about the task instructions.

#### Automated reading span (complex WM capacity) [34]

Participants were presented with alternating letters and sentences, and asked to remember the letters while assessing whether each sentence was sensical. Set sizes and scoring were identical to the Automated Operation Span.

#### Combined span task (complex WM capacity)

Scores from the Automated Operation Span and Automated Reading Span were summed to create a single measure estimating a participant’s complex working memory capacity.

### Far Transfer – Standardized Intelligence Tasks

#### Raven’s advanced progressive matrices (fluid intelligence) [35]

Each item presented a three-by-three grid filled with patterns, with the bottom-right entry missing. Participants selected the best of 8 choices to fill the missing location based on the pattern of the other elements in the matrix. We created two forms of the 36-item RAPM test for pre- and post-testing. The two forms were equated for difficulty based primarily on published accuracy rates per item [35] and secondarily on pilot experiments assessing the average response time per item. Because the last two items of the test are much more difficult than the rest of the test and are not matched to each other in difficulty, the dependent variable was the number of correct responses out of the first 17 items. Form A consisted of items 1, 3, 5, 7, 9, 11, 13, 15, 17, 19, 22, 23, 25, 28, 29, 31, 34, and 36, while form B consisted of items 2, 4, 6, 8, 10, 12, 14, 16, 18, 20, 21, 24, 26, 27, 30, 32, 33, and 35. Participants were given 25 minutes to complete each half of the RAPM.

#### Wechsler Abbreviated Scale of Intelligence (WASI)/Wechsler Adult Intelligence Score III (WAIS-III) [31], [36]

The WASI and WAIS are commonly used assessments of standardized intelligence. Because they have been normed against each other and use common sub-tests with different forms, the two tests provide a simple way of acquiring a matched IQ measurement before and after training. Rather than counter-balancing the two tests, the WASI was administered pre-training and the WAIS post-training so as to maximize the sensitivity of measuring any training-related transfer between groups.

#### WASI/WAIS blocks

Participants were given a set of physical blocks with red and white shading on them, and asked to assemble them so as to replicate a target pattern. The amount of time needed to replicate the patterns was the raw score that was converted into a scaled score, which was then used as the dependent measurement.

#### WASI/WAIS matrices

Participants selected the best-fitting item to complete a grid of figures, based on abstract rules and relations between the other figures in the grid.

#### WASI/WAIS vocabulary

Participants were required to verbally define progressively more challenging vocabulary words.

#### WASI/WAIS similarities

Participants were asked to relate pairs of concepts (e.g., How are a snake and an alligator alike?).

### Far Transfer – Reading Comprehension

#### Nelson denny comprehension subtest [37]

Participants were asked to read five short passages and respond to 38 short questions about the contents of those passages.

#### Nelson denny reading rate [37]

During the first passage in the comprehension subtest, participants’ reading rate was assessed by recording the number of words read in the first minute.

### Far Transfer – Speed of Processing

#### WAIS-III digit/symbol coding [36]

Participants were provided with a set of digit-symbol pairs and a list of digits. Under each digit, participants wrote down as many corresponding symbols as possible during a two-minute span.

#### Woodcock-johnson III tests of cognitive abilities: visual matching [38]

For 3 minutes, participants scanned rows of numbers and circled the two identical numbers in that row.

#### Woodcock-johnson III tests of cognitive abilities: pair cancellation [38]

For 3 minutes, participants scanned rows of figures and circled each instance in which a target picture was followed immediately by a second target picture (e.g., a cat followed by a tree).

### Personality Measurements

#### Dweck intelligence questionnaire [39]

Participants were asked to indicate the extent that they agree/disagree with 8 statements regarding the malleability of intelligence (e.g., “You have a certain amount of intelligence, and you really can’t do much to change it”) on a 5-point scale. The dependent measure is the sum of their answers (with some items reversed in scoring), with a lower score indicating a more static view of intelligence.

#### Conscientiousness factor questionnaire [27]

Participants were asked to rate how well-described they were by 12 statements assessing their perception of their own conscientiousness on a 5-point scale (e.g., “I strive for excellence in everything I do.”). The statements were taken from the Conscientiousness section of the NEO-FFI. The dependent measure is the sum of answers (with some items reversed in scoring), with a lower score indicating a self-perception as less conscientious.

#### Short grit scale [40]

Participants were asked to rate how well-described they were by 8 statements assessing their perception of their own “grit” on a 5-point scale (e.g., “I am diligent.”). The dependent measure is the sum of their answers (with some items reversed in scoring), with a lower score indicating a lower self-perception of grit.

### Training Protocols

For both the dual n-back and MOT groups, training sessions lasted approximately forty minutes per day, and participants were asked to commit to one training session per day, Monday through Friday, at a consistent time. In the event that a training session was missed, participants were allowed to train on the weekend, or to train twice in one day, so long as the two sessions were separated by at least three hours of time. This option was used by 3 of the MOT participants (with a maximum of 3 double-session days) and 6 of the n-back participants (one subject had double-sessions on 5 days in an attempt to complete the experiment before winter break, the other five had a maximum of two double-sessions). Participants in the dual n-back training group completed 20 sessions in an average of 29.2 days (min 21 days, max 42 days), while participants in the MOT training group completed 20 sessions in an average of 28.6 days (min 23 days, max 37 days).

In addition to the weekly bonus payment for completing all five sessions in that week, participants were emailed on a weekly basis congratulating them on their attendance, alerting them of their bonus, and informing them of the progress they had made in training that week. This email was intended to be motivational, so the email highlighted new achievements from the previous week (e.g., a new peak in a performance measure).

#### Multiple Object Tracking (MOT) training

Participants assigned to the MOT task performed 90 adaptive tracking trials per day, as described in the baseline MOT testing session. (Due to experimenter error, three MOT participants had some days of training with 60 trials instead of 90 trials. These days were during the first half of the training period, and no subject had more than three short days.) The initial speed of the tracked objects was determined by the final speed of the pre-training baseline MOT session, which was reached via the staircasing procedure described above. On subsequent days, the first trial’s speed was set to the speed of the last trial on the previous training day. The speed of the tracked objects was adjusted upward by.5 degrees of visual angle/second whenever two consecutive trials were answered correctly and downward by.5 degrees/second when two consecutive trials were missed. Participants were allowed to take breaks, as needed.

#### Dual n-back training

Participants assigned to the dual n-back training group performed 30 blocks of the task per session, as described above. Due to evidence for a dose-dependent relation between the amount of dual n-back training and gains in transfer to fluid intelligence in Jaeggi et al., 2008 [6], we provided all participants with more training (30 blocks/session) than the highest level of training in that study (20 blocks/session) to maximize the dose of training received and to increase the likelihood that the WM training would yield near- and far-transfer gains.

The manner in which the difficulty adapted followed the task described in Jaeggi, et al, 2008: If the participant made more than 5 errors in a block, the *n* of the next block was decreased by 1, to a minimum of a 1-back block. If the participant made 2 or fewer errors in both the auditory and visual n-back stream, the *n* of the next block was increased by 1, with no maximum *n-back* level. In all other cases, the difficulty level remained the same. All participants started at a 2-back level on day 1, and on later days their starting difficulty was set to be the same as the last block on the previous day. Participants were encouraged to take short breaks, as needed, to stay focused during training.

Analyses of the pre- and post-training n-back measures and of the training n-back sessions had to be conducted with different dependent measures. The pre- and post-training measures were analyzed with accuracy (d’) because loads were held constant. The training sessions could be not analyzed in this way because load was adaptively altered to keep accuracy as constant as possible. Therefore, training session measures were analyzed with load as the dependent measure.

## Results

### Initial Group Comparisons

One-way ANOVAs confirmed that the groups did not significantly differ on any of the behavioral measurements (all p’s >.19 for 3-group comparison, all p’s >.19 for comparison between the two active training groups).

### Initial Task Correlations

One plausible reason to expect transfer from a trained task to an untrained task is that those tasks share common cognitive or neural processes, as evidenced by high correlations between those tasks. The full set of correlations between initial scores on the behavioral outcome measures and initial scores on the training tasks is displayed in Table 2. Of particular importance for the hypothesized transfer from the dual n-back task to more general cognition, a participants’ initial performance on the dual n-back task (as measured by d’ across the 2-back to 6-back difficulty levels) was significantly correlated with fluid intelligence measures (Ravens Advanced Progressive Matrices and the WASI Matrix Task), complex working memory measures (Operation Span, Reading Span, and their combined score), and a reading comprehension measure (Nelson-Denny Reading Comprehension). In contrast, the adaptive control task (MOT) did not show significant correlations with those measures.

### Trained Tasks

**Figure 1:**
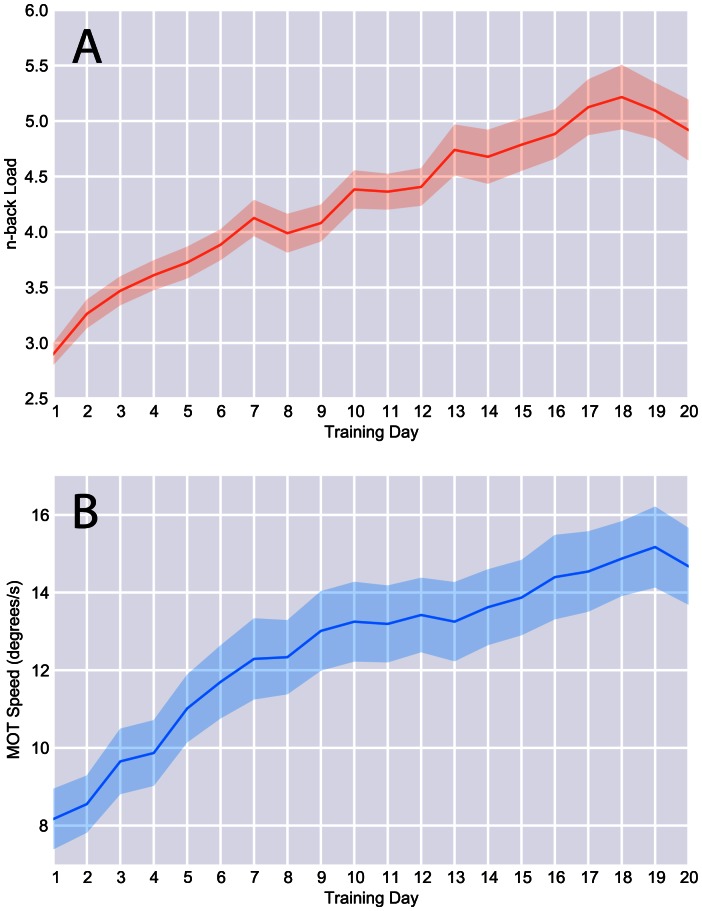
Performance across training sessions. **A)** Mean dual n-back load and B) mean multiple object tracking speeds achieved per session of training are displayed. Shaded area represents standard error of the mean.

Both active training groups improved significantly with practice on their trained task (Figure 1). In the dual n-back training condition, participants improved from an average n-back of 3.19 (SD = .47) over the first three days of training to an average n-back of 5.1 (SD = 1.1) across the last three days of training [t(19) = 9.70, p<.0001]. All 20 dual n-back participants improved substantially, with everyone completing at least one dual 5-back block, 17 participants completing a dual 6-back block, 12 participants completing a dual 7-back, 6 participants completing a dual 8-back, and 3 participants completing one or more dual 9-back blocks. (Figure S1 shows individual training gains for both the MOT and dual n-back groups.).

Some participants reported changing their strategies for performing the dual n-back task throughout training. The most commonly reported strategy was mentally superimposing the auditorily presented letter in the visually presented spatial location in an attempt to consolidate the two input streams, though this was not a universally reported strategy. However, the improvement observed on this task cannot be explained merely by strategy shifts. Participants reported fixing on their own idiosyncratic strategies during the first few days of training, and continued making improvements over the course of the 20-day training period long after their particular strategy was chosen.

In the MOT training condition, participants improved from an average tracking speed of 8.8 degrees/second (SD = 3.2) over the first three days of training to an average speed of 14.9 degrees/second (SD = 4.2) over the last three days. [t(18) = 11.6, p<.0001] Although there was a range of improvement in this condition, all participants were able to track items at least 12 degrees/second at some point during their training, with six participants becoming able to track 4 targets moving at faster than 20 degrees/second.

Some MOT participants also reported changing strategies early in the course of training, although these participants tended to fixate on a strategy early in training and then continued using it throughout the remainder of the training period. Strategies varied widely, with the most commonly reported three strategies being (1) to visualize the tracked dots as corners of a quadrilateral, (2) to attempt to track the center of mass of the four target dots, or (3) to remain fixated on the center fixation cross and track all four target dots in the periphery, without trying to merge the targets into a coherent single object.

Improvements on both the n-back and MOT tasks were specific to their training group. Comparing performance on these two tasks during the behavioral testing before and after training reveals a double-dissociation between the groups – the MOT training group improved on the pre- and post-training MOT task significantly more than did either the passive control or the n-back group [Group×Time interaction, F(2,117) = 37.7, p<.0001], while the n-back group improved on the n-back task significantly more than either the passive control or the MOT training groups [Group×Time interaction, F(2,117) = 47.3, p<.0001]. Direct comparison of the two training groups with the No-Contact group revealed whether either training group exhibited any transfer to the untrained task. The MOT group exhibited no more gain on the dual n-back task than the No-Contact group [Group×Time interaction, t(55) = .17, p = .86], and the dual n-back group exhibited no more gain on the MOT task than the No-Contact group [Group×Time interaction, t(54) = .56, p = .57).

### Duration of Training Gain

**Figure 2:**
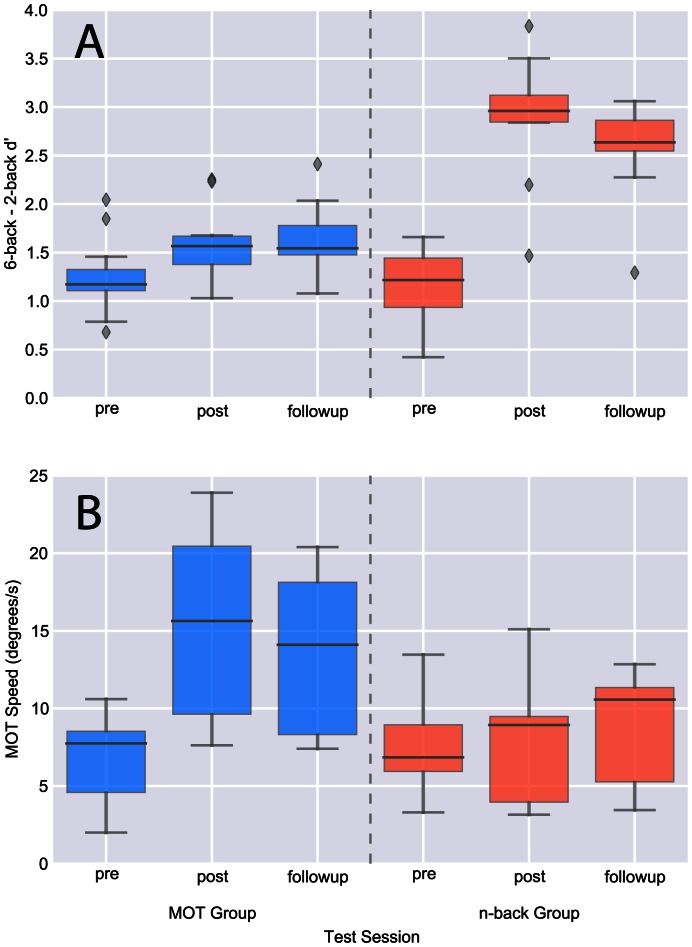
Duration of training effects. **A)** Difference between dual 6-back d' and dual 2-back d’ is shown for pre-training, post-training, and six-month follow-up sessions for both active training groups. **B)** Multiple object tracking speed is shown at all three time points for both active training groups. Solid dark, horizontal line indicates condition median; filled areas encode middle 50%. Whiskers extend 1.5 times the interquartile range beyond the box bounds.

Gains made on a trained task largely persisted for 6 or more months past the end of training (Figure 2). Comparing the d’ measurement for the 2-back through 6-back tests for the group of participants who received n-back training, paired t-tests showed significant improvements from pre-training testing (M: 1.23, SD:.42) to post-training testing (M: 2.92, SD:.67), [t(9) = 7.49,p<.0001], and from pre-training testing to follow-up testing (M: 2.60, SD:.53) [t(9) = 7.96, p<.0001]; there was also a significant decrease from post-training testing to follow-up testing [t(9) = 3.57, p<.01]. In comparison, the MOT training group showed a smaller gain from pre-training testing (M: 1.25, SD:.40) to post-training testing (M: 1.59, SD:.39) [t(10) = 4.96, p<.001], but no significant difference between the post-training testing and the follow-up testing (M: 1.64, SD:.36) [t(10) = .96, p>.35].

For the MOT speed assessment, a similar pattern of enduring skill emerged (Figure 2). The MOT training group showed significant improvements from pre-training testing (M: 6.60, SD: 2.87) to post-training testing (M: 15.43, SD: 5.92) [t(10) = 8.03, p<.0001], and from pre-training testing to follow-up testing (M: 13.75, SD: 5.08) [t(10) = 9.27, p<.0001]; there also was a significant decrease from post-training testing to follow-up testing [t(10) = 3.13, p<.05]. In comparison, the n-back training group did not show a significant gain from pre-training testing (M: 7.20, SD: 3.1) to post-training testing (M: 8.37, SD: 3.82) [t(8) = 1.43, p>.18], or from pre-training testing to follow-up testing (M: 8.87, SD: 3.45) [t(8) = 1.46, p>.18]. The post-training testing was also not significantly different than the follow-up testing for the n-back training group [t(9) = 1.02, p>.33].

### Transfer Tasks

In contrast to the substantial improvements seen on the trained tasks, participants did not generally show improvements on the tasks measuring near or far transfer (Table 3). The one statistically significant improvement (although it did not survive Bonferroni correction for multiple comparisons) was on the Matrix Reasoning section of the Wechsler tests, where the MOT group showed an average improvement of 2 items that was not observed in the n-back training group.

### Power Analyses

To assess whether the observed lack of transfer was a result of an underpowered sample size, we used the G*Power software package [41] to assess the sensitivity of the behavioral tests. The final column of Table 3 reports the minimum effect size that could be detected in a between-groups interaction, based on the sample sizes in this study and the correlation between the pre- and post-testing scores for each test. (This test uses the correlation between the pre- and post-training scores in the passive control group as a measure of test-retest reliability. The more consistent the relationship between the two scores is, the smaller the detectable change will be.) The sensitivity level was set at p<.05 for failing to observe a real effect. For every transfer measure, this experiment had sufficient power to detect a medium (*f* = .25) to large (*f = *.40) effect, and had ample power to detect the effect sizes reported in the initial Jaeggi experiment (*d = *.68).

### Correlations between Training Improvement and Transfer

Some prior research has observed transfer gains in only those participants who successfully improved on the trained task [15], [42]. We therefore performed several analyses to examine whether there were individual differences among participants that were associated with either training gains or with transfer from training to other measures. For this purpose, a training improvement score was calculated for each participant by subtracting the average performance during the initial three days of training from the average performance during the last three days of training. For the n-back training group, the average “n” of the n-back blocks was calculated, whereas for the MOT training group, the average object movement speed was calculated.

One method of assessing whether the amount of training improvement affects the degree of transfer is to measure the correlation between training and transfer gains. For both the n-back and MOT groups, a positive correlation was observed between the amount of improvement during training and the amount of improvement on the trained task between the pre- and post-assessment (n-back r = .85, p<.0001; MOT r = .77, p<.0001). However, the amount of training gain did not significantly predict improvement on any transfer task; participants who improved to a greater extent on the training tasks did not improve more or less on potential transfer tasks than did participants who improved to a lesser extent (all n-back r values <.33, all p’s >.15; all MOT r values <.38, all p’s >.11). Figure S2 depicts the absence of a relation between improvement on trained tasks and the post-training changes in the RAPM and the combined span tasks.

Another analysis that has previously revealed a difference in transfer between participants who exhibited larger or smaller training gains has been a division of participants into groups based on training gains above or below the group median (median split) [15]. Such a median split of participants in the present study who performed the n-back training yielded no significant differences in transfer between groups (all n-back t-ratios <1.78, all p’s >.09). The only transfer measure that approached significance (at p = .09) was on the RAPM test, in which the participants who improved *less* on the trained n-back task had higher scores on the post-training behavioral testing. Similarly, when separating the MOT participants into two groups based on median MOT improvement, the two groups showed no significant differences in transfer performance (all MOT t-ratios <1.74, all p’s >.10).

A clustering algorithm (an example of which is the k-means algorithm 43) is another approach to classifying participants into two groups based on differences in training gains, and this approach has shown that participants classified as responding to training show gains on transfer tasks, whereas participants classified as not responding to training fail to show gains on transfer tasks [42]. Clustering algorithms have the advantage of classifying different numbers of participants into responder and non-responder groups when such a division does not occur naturally at the median. The clustering algorithm applied to the present n-back training data yielded two clusters, one with 9 participants and the other with 11 participants, which was a close approximation of the median split grouping that had yielded 10 participants in each group, and again there were no significant differences revealed in the transfer measures between groups (all t-ratios <1.50, all p’s >.15).

Clustering algorithms, however, do not always yield meaningful or easily interpretable clusters. The same clustering algorithm applied to the MOT training data yielded clusters with 14 “non-responder” participants and 5 “responder” participants, although the average participant in the “non-responder” cluster increased their MOT tracking speed by more than 5 degrees/second.

### Correlations between Pre-training Measurements and Training Gains

No pre-training behavioral score significantly predicted the amount of task improvement during training in either the n-back or the MOT group (all n-back r values <.31, all p’s >.17; all MOT r values <.34, all p’s >.16).

### Correlations of Personality Measurements and Transfer

We also examined whether personality assessments were associated with different training or transfer outcomes. Neither the Dweck measure of attitude toward intelligence (a “growth mindset”) nor measures of conscientiousness or grit correlated significantly with training gains on either training task, although there was a trend toward a significant negative correlation between the growth mindset and improvement on the n-back training task (r = −.44, p = .051), such that participants who viewed intelligence as more malleable had less improvement across their n-back training. A greater growth mindset score was positively correlated, however, with improvement on the Ravens Advanced Progressive Matrices in the n-back group (r = .53, p = .017) and in the passive control group (r = .51, p = .027), but not in the MOT control group (r = .031, p>.9). No other transfer measures were significantly predicted by growth mindset scores.

Although the conscientiousness scores and “grit” scores were highly correlated in each of the three treatment groups (n-back r = .75, p<.001; MOT r = .70, p<.001; passive r = .76, p<.001), the two measures differed in their correlations with the behavioral outcome measures. A higher “grit” score predicted less improvement on the RAPM for the n-back group (r = −.45, p = .049) and the MOT group (r = −.58, p = .009), such that participants who viewed themselves as having more “grit” improved less on the RAPM after training, although this relationship did not hold for the No-Contact group (r = .17, p = .5). Similarly, a higher score on the conscientiousness measure predicted less improvement on the RAPM for the MOT group (r = −.57, p = .01), such that participants who saw themselves as more conscientious improved less on the RAPM after training, although this was not observed in either of the other two groups (n-back r = −.21, p = .37; no-contact r = −.04, p = .85). Finally, a high conscientiousness score predicted a lower Pair Cancellation improvement within the MOT group (r = −.47, p = .04), but not in the n-back or no-contact control groups (n-back r = −.07, p = .77; no-contact r = −.13, p = .58). No other transfer measures were significantly predicted by either conscientiousness or grit scores.

## Discussion

This experiment yielded one major finding and some new observations. The major finding was a failure to observe any gains in measured fluid intelligence after working memory training. Although participants improved substantially on their trained tasks, neither WM training nor multiple object tracking training provided benefits on speed of processing tasks, other standardized measures of intelligence, or measurements of reading comprehension. The lack of transfer from WM training to other measures occurred for both near-transfer tasks (other complex working memory tests) and far-transfer tasks (e.g., fluid intelligence measures) and was relative both to an active control training group (MOT training) and a no-contact control group. The absence of transfer occurred despite robust learning on the trained tasks and substantial retention of those acquired skills lasting over six months.

### Magnitude of Training Effects

Critically, the amount of improvement seen on the dual n-back task was nearly identical to the amount of training improvement seen in the prior study reporting improvements in fluid intelligence [6]. In the previous report, participants initially were able to perform a dual 3-back task, and ultimately averaged slightly better than a dual 5-back task after 19 days of training. In the present experiment, participants’ average performance across the first three days was 3.19-back, and average performance across the last three days was 5.19 back. Participants in previous attempts to replicate the original Jaeggi finding exhibited lesser amounts of dual n-back improvement across training. Specifically, participants achieved an average dual n-back level of approximately 4.0 in one study [14], and an average of approximately 4.1 in another study [13]. The somewhat lower final levels of WM performance in these two failed replications left open the possibility that the discrepent findings on transfer to fluid intelligence were related to the level of WM capacity learned through training. The present WM training outcomes, which closely resemble those from Jaeggi et al., 2008, indicate that failure of transfer to other measures of cognition and fluid intelligence cannot be accounted for either by gains in trained WM performance or in final level of WM performance.

Although participants in the present study had 33% more training per session than the those in the study from Jaeggi et al, 2008, they did not exhibit greater gains in WM capacity than those who had an equal number of sessions in the Jaeggi et al., 2008, study. It is not clear why the additional training did not yield additional WM capacity. One possibility is that there is a limit or asympote to dual n-back training. Another possibility is that participants vary in the rate of WM training gains, which could be related also to transfer.

The amount of improvement on the active-control MOT task was comparable with the amount of improvement observed in the dual n-back group. Participants, on average, improved their initial score by 1.59x in the dual n-back condition, while participants trained in the MOT condition improved their initial score by 1.69x, from an initial 3-day average of 8.8 degrees per second to a final 3-day average of 14.9 degrees per second. This comparable level of improvement validates the use of the MOT task as a suitable active control for the dual n-back task. Although the MOT task has been widely used to study visuospatial WM capacity (e.g., [33]), this is the first study to show that MOT skill can be acquired and maintained over a long period.

### Specificity and Duration of Training Effects

Training in both active groups was robust and specific to the type of training in that participants who were trained on the one task exhibited substantial gains on the trained tasks, but no gains on the other task. The duration of sustained improvement from dual n-back or MOT training, however, has been previously unknown in healthy young adults. In this experiment, 18 participants returned after their 20 days of training to assess the longevity of their specific training gains. Both the MOT and n-back groups showed significant improvement from their pre-testing to post-testing scores, and those improvements were largely, although not completely, maintained 6 months later. Although we failed to observe improvements in fluid intelligence in this experiment, the maintenance of the training improvements, despite 6 months without further training, seems to be a necessary component of any working memory training paradigm aimed at creating enduring improvements.

### Transfer – General Expectations

Transfer from a trained task to an untrained task is expected when the two tasks share common components, whether they be cognitive processing steps or reliance on similar neural activations [44]. In *near-transfer* tasks, the trained task bears surface similarities to the target task, such that observed improvements on the target task could conceptually be the result of either a learned strategy during training that is also applicable to the transfer task, or the result of actually improving an underlying cognitive skill. In *far-transfer* tasks, the demands of the task do not involve an overt shared strategy, so there are fewer mechanisms for training in one task to produce benefits on the second. Although experiments examining transfer from the dual n-back task to fluid intelligence (*far transfer*) have reported mixed results [6], [13]–[15], [17], [18], some WM training studies report transfer from the trained WM task to another untrained WM task *(near transfer*) (e.g., [21], [22]), including one study in which dual n-back training similar to that used here resulted in improved operation span performance [20].

### Near Transfer

In the present experiment, the two tasks most conceptually similar to the dual n-back training were the Operation Span and Reading Span tasks that, like the dual n-back task, are tasks of complex working memory (CWM). The finding that performance on all three CWM tasks was significantly correlated supports the idea that the three tasks share underlying mechanisms. Previous experiments training WM tasks have sometimes shown transfer to non-trained WM tasks (e.g., [20]–[22]). In this study, however, there was no evidence of transfer from the dual n-back task to the Operation or Reading Span tasks alone or in combination, or in relation to the amount of learning on the n-back task.

### Far Transfer - Fluid Intelligence

The possibility that WM training would enhance fluid intelligence is supported by behavioral findings reporting high correlations between complex WM scores and fluid intelligence scores [9], [45], which indicates shared psychological mechanisms, and neuroimaging findings reporting similar activations for complex WM and fluid intelligence tasks, which indicates shared neural mechanisms [9], [46]–[48]. Indeed, we also observed strong correlations between initial performance on the dual n-back task and two measures of fluid intelligence. This relationship was specific – there was no correlation between initial performance on the MOT task and the same measures of fluid intelligence.

There was not, however, any improvement on the fluid intelligence tasks after dual n-back training compared to either the active control group or the passive control group. There was also no relation between the amount of improvement on the dual n-back task and transfer to either fluid intelligence measure. Thus, although it appears that the necessary conditions for transfer to occur were achieved in the experiment, there was no evidence of transfer from WM training to fluid intelligence measures.

There was one significant transfer effect from training to a fluid intelligence measure: MOT training improved performance on the matrix reasoning section of the Weschler Intelligence Tests. Although this finding may be of interest, there are two reasons to suspect it could be spurious. First, the group who received MOT training showed no more improvement in the matrix reasoning score than did the no-contact control group. Second, the improvement by the MOT-trained group did not extend to the other matrix-based fluid intelligence measure, the Ravens’ Advanced Progressive Matrices. For these reasons, as well as the absence of any behavioral correlation between initial MOT performance and either measure of fluid reasoning, it seems more likely that this was an example of the sort of false positive finding that can occur with so many behavioral measures, rather than genuine transfer from MOT training to fluid intelligence.

### Far Transfer – Other Tasks

WM training has sometimes been reported to yield transfer to other kinds of performance, including improvements in domains of cognitive control that are often associated directly with WM, such as attentional control (e.g., Stroop task) and reading comprehension [12], among others (reviewed in [49]). These findings motivated inclusion of additional measures, including the specific test of reading comprehension that demonstrated benefit from WM training [12] and processing speed (which typically correlates with WM capacity) [50]. We did not, however, observe transfer from either WM or MOT training on any of these measures.

### Working Memory in the Multiple Object Tracking Task

The MOT task was conceptualized as a control training task involving perceptual skill learning, but learning on the MOT task could alternatively be conceptualized as training of visuospatial working memory. The fact that pre-training MOT performance did not correlate with complex working memory tasks, matrix reasoning tasks, or reading comprehension measures indicates that if MOT involves working memory, it may selectively involve the visuospatial component and not executive or phonological components. Further, the substantial gains on MOT performance did not produce transfer to other tasks, with the exception of a single isolated measure.

### Personality Measurements and Motivation

There is evidence that personality factors can modulate the influence of WM training on gains in fluid intelligence [17], and we examined personality factors that could, in theory, influence such transfer. One study found that greater conscientiousness predicted higher levels of performance during training on single n-back tasks, although it did not predict performance in a separate group using dual n-back training [17]. Furthermore, across both n-back training groups, conscientiousness was negatively correlated with fluid intelligence gains. In contrast, we did not observe a correlation between conscientiousness (or the highly correlated “grit” scores) and performance during either dual n-back training or MOT training. Similar to the prior findings, higher conscientiousness scores predicted smaller improvements on the Ravens Advanced Progressive Matrices (RAPM) in the MOT training group, while higher grit predicted smaller improvements on the RAPM in both the dual n-back and MOT training groups. We fail to support their broader claim that conscientiousness negatively predicts transfer to fluid intelligence, however, as neither conscientiousness nor grit scores predicted change in the performance IQ measures of the WASI/WAIS test (the matrix reasoning task and block design measures) that load highly on fluid intelligence.

Another plausible variable affecting transfer from WM training to fluid intelligence is the participant’s attitude about intelligence. In some studies, students who believe that intelligence is a malleable trait that can be enhanced by effort (i.e., student who have a “growth mindset”) show greater learning than students who believe that intelligence is a fixed trait [30]. This study did not show that pattern. Instead, we observed a trend in the opposite direction for the n-back training group – participants who viewed intelligence as fixed improved more over the course of n-back training than did participants with a growth mindset toward intelligence. We observed no relation between attitudes toward intelligence and improvement on multiple object tracking.

There were also some relations between growth mindset and improvement on the RAPM. Participants with greater growth mindsets in the n-back group exhibited greater growth on RAPM scores. Although this could be interpreted as revealing that greater growth mindset facilitates greater transfer of working memory training to fluid intelligence, two other findings contradict this interpretation. First, greater growth mindset was not related to gains on the other fluid intelligence measure or on other working memory tasks. Second, the same relation between greater growth mindset and greater growth on RAPM scores was observed in the No-Contact group who had received no training. Personality did not seem to account for variation in transfer from WM to other kinds of cognitive ability.

A more general measurement of motivation is difficult to obtain. It is possible that we did not observe the same benefit of WM training for fluid intelligence as previous groups because our participants were somehow less motivated to improve. Subjectively, participants appeared excited about the prospect of transfer from WM training to other aspects of their life, especially academic endeavors. Certainly, every effort was made to motivate the trained participants in both the dual n-back training and the active control groups through several means: 1) by explicitly telling both groups that the task could possibly make them “smarter”, 2) by providing weekly encouraging e-mails highlighting their accomplishments, and 3) by providing monetary bonuses for conscientious training. Although it is unknown how effective these manipulations were, the overall amount of training improvement seen in this study was nearly identical to that seen in the original Jaeggi study, providing at least an indirect confirmation of similarly motivated participants.

### Sensitivity to Detect Transfer

Failure to observe transfer could reflect insufficient statistical power, but for several reasons it appears that this unlikely to explain the lack of transfer observed in the present study. First, there were almost no transfer effects in the training groups that numerically surpassed the simple test/retest practice effects exhibited by the no-contact control group. Second, the effect size in the initial report of transfer from WM training to fluid intelligence was substantial (Cohen’s d = .65) [6]. The sample in the present study would have allowed detection of transfer with an effect size of *d* = .27 or better. In the social sciences, a “small” effect size for an independent means t-test (which is the statistic that the interaction of a repeated measures ANOVA evaluates) is regarded as *d* = .2, while a “medium” effect size on this test is *d = *.5 [51]. Therefore, the present study ought to have had sufficient power to replicate the initial report and to find most small effects, although it may have been underpowered to detect very small differences between training groups.

### Implications for Working Memory Training

The goal of enhancing core cognitive abilities that support and constrain performance in many cognitive domains is an important educational and clinical goal, and speaks to basic theoretical interests about plasticity of the human mind and brain. For these reasons, the report that WM training enhances fluid intelligence [6] has generated great interest for many researchers in human psychology and human cognitive neuroscience as well as in the public at large. The promise of such training for enhancing the cognitive capacity of the human mind has been supported by other studies reporting WM training benefits on reading comprehension [12], [52], mathematical ability [53], and ADHD symptomology [54], and some training programs have even gone so far as to show the neural changes occurring with WM training that theoretically enable the transfer to other domains [55]–[57].

However, several other studies have failed to observe any transfer from WM training to broader cognitive functions. In one well-publicized finding, for example, 11,430 people in the UK performed a variety of on-line cognitive training tasks at home for a 6-week period, and although improvements were found on all trained tasks, there was no near or far transfer to any untrained task [58]. Reviews and systematic meta-analyses also do not conclude that WM training generally enhances broad cognitive abilities [49], [59].

Broad reviews and discussions about cognitive training often intertwine several distinct issues, such as whether WM training is helpful for young adults, children, older adults with typical age-associated cognitive losses, or patients with diagnoses such as ADHD. The present study, however, focused specifically on the possibility that dual n-back training, if effectively delivered as indexed by gains in WM, could enhance fluid intelligence as reported by Jaeggi et al., 2008. Three published studies, including the present study, have attempted to replicate that finding without success. One study included both an adaptive active control group and multiple measures of both near transfer (WM measures) and far transfer (fluid intelligence, crystallized intelligence, and processing speed measures [14]). Like the present study, that research found substantial learning on the trained WM capacity task, but no near transfer to other WM tasks or far transfer to fluid intelligence, crystallized intelligence, and processing speed. Another study, without an adaptive control group, also failed to find such transfer [13].

It would be valuable to discern the factors across studies that are associated with success or failure in having WM training improve fundamental faculties of the human mind as measured by improved performance on a range of untrained tasks. The present study indicates that the amount of WM training does not appear to account for such variability in outcomes, because the present study involved more training than that reported in a study with positive findings [6]. Some studies have found that greater gains in training were associated with transfer (e.g., [15], [42]), but we did not observe any such relation between training gains and transfer in three independent analyses. Variation in personality could be another factor [17], but personality measures of conscientiousness, grit, or attitudes towards intelligence did not correlate with training transfer in the present study. It is difficult at present to identify any one factor across studies that plausibly explains transfer success.

Besides individual differences among participants, another important factor related to transfer gains may be the nature of training program. The present study trained participant on one of two homogenous tasks, the n-back or MOT task. An alternative approach is to employ a training program that involves multiple, heterogeneous cognitive training tasks. Such heterogeneous training has yielded transfer gains in some WM training studies (e.g., [16], [42]). Heterogeneous training may have the advantage of training multiple specific cognitive skills that initially vary across individuals and that promote transfer to heterogeneous transfer tasks that vary in their specific cognitive demands.

It is possible that WM training may be more consistently beneficial for individuals performing suboptimally, rather than the high-performing young adults who have been the participants in the above reviewed studies. There are reports of successful transfer of WM training in patient groups with ADHD [54], [60] or stroke [61], or in particularly younger or older populations (e.g., [21], [53]). It is also these groups of individuals for whom effective cognitive training may be most helpful in improving everyday functioning. Future research will, hopefully, reveal principles by which the effectiveness of cognitive training programs, beyond gains on the trained program itself, can be predicted.

## Supporting Information

Figure S1 **Individual Subject Training Gains.** Beginning and ending dual n-back loads/Multiple Object Tracking (MOT) speeds are presented for each participant. Beginning points represent the average performance across the first three days of training, while ending points display the average performance across the final three days of training.
(PDF)

Figure S2 **Relationships Between Training Gains and Transfer Measures. A)** Correlation between improvement on the dual n-back task during training and the difference between pre- and post-training Ravens Advanced Progressive Matrices (RAPM) scores. **B)** Correlation between dual n-back improvement and change on the Composite Span Task scores. **C)** Correlation between improvement in Multiple Object Tracking (MOT) speed and RAPM change. **D)** Correlation between MOT gains and Composite Span Task score changes. All p’s >.05. Error bands are bootstrapped 95% confidence intervals for the regression.
(PDF)
