# Supplementary figures and images for: Failure of Working Memory Training to Enhance Cognition or Intelligence
Source: PLoS One. 2013 May 22;8(5):e63614. doi: 10.1371/journal.pone.0063614 (PMC3661602; doi:10.1371/journal.pone.0063614)

### Dual n-back Training Gain

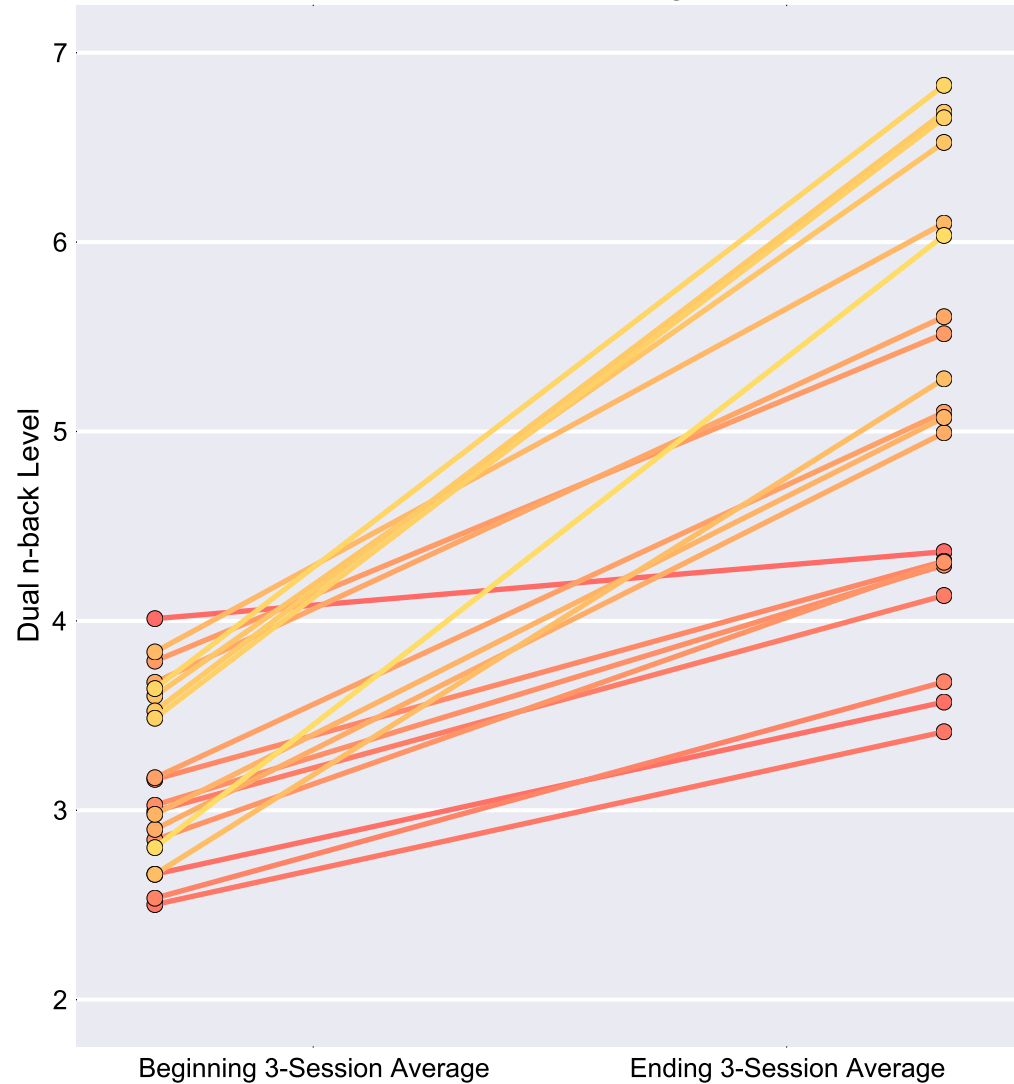

### MOT Training Gain

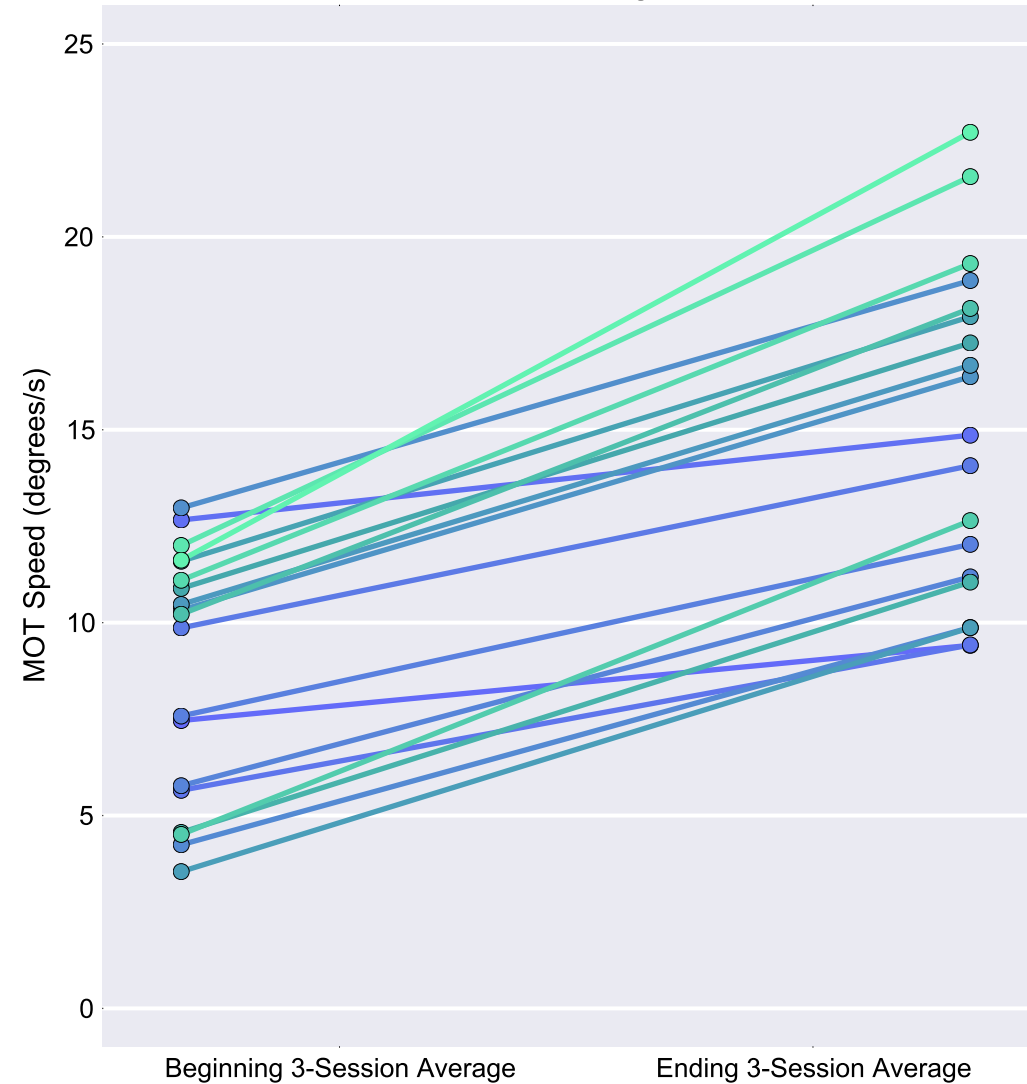

Supplement: Figure S1 — Individual Subject Training Gains. Beginning and ending dual n-back loads/Multiple Object Tracking (MOT) speeds are presented for each participant. Beginning points represent the average performance across the first three days of training, while ending points display the average performance across the final three days of training. (PDF) [file pone.0063614.s001.pdf]

**A**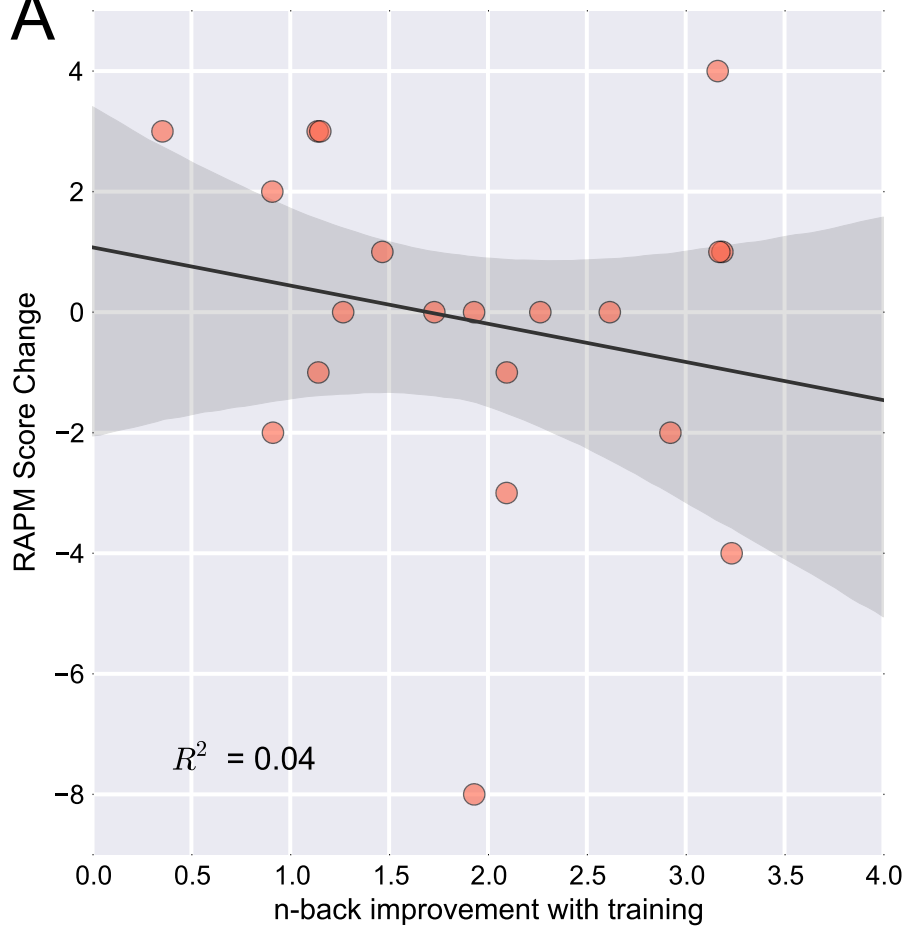**B**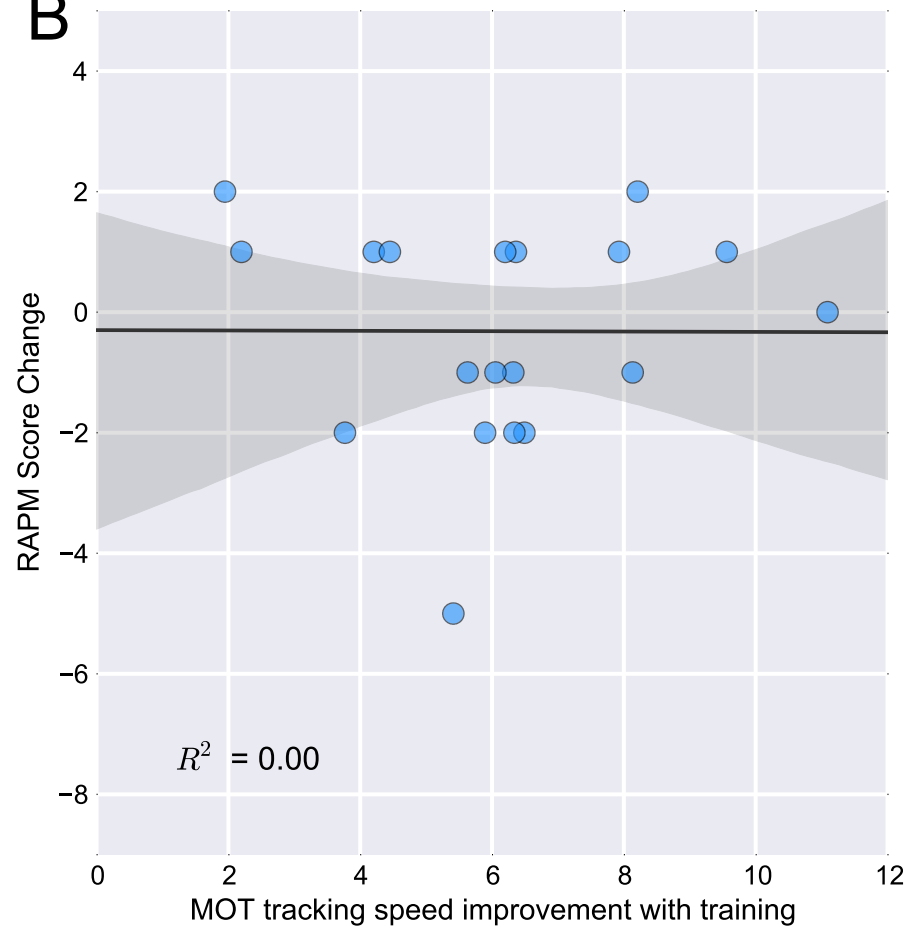**C**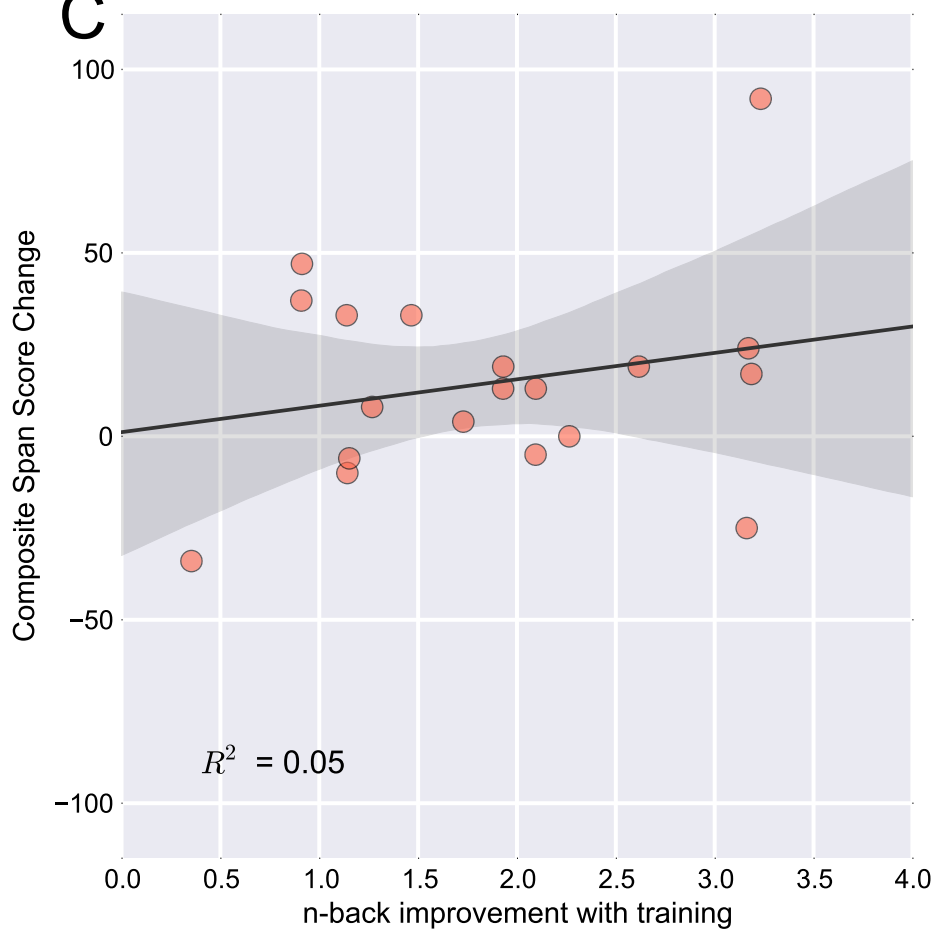**D**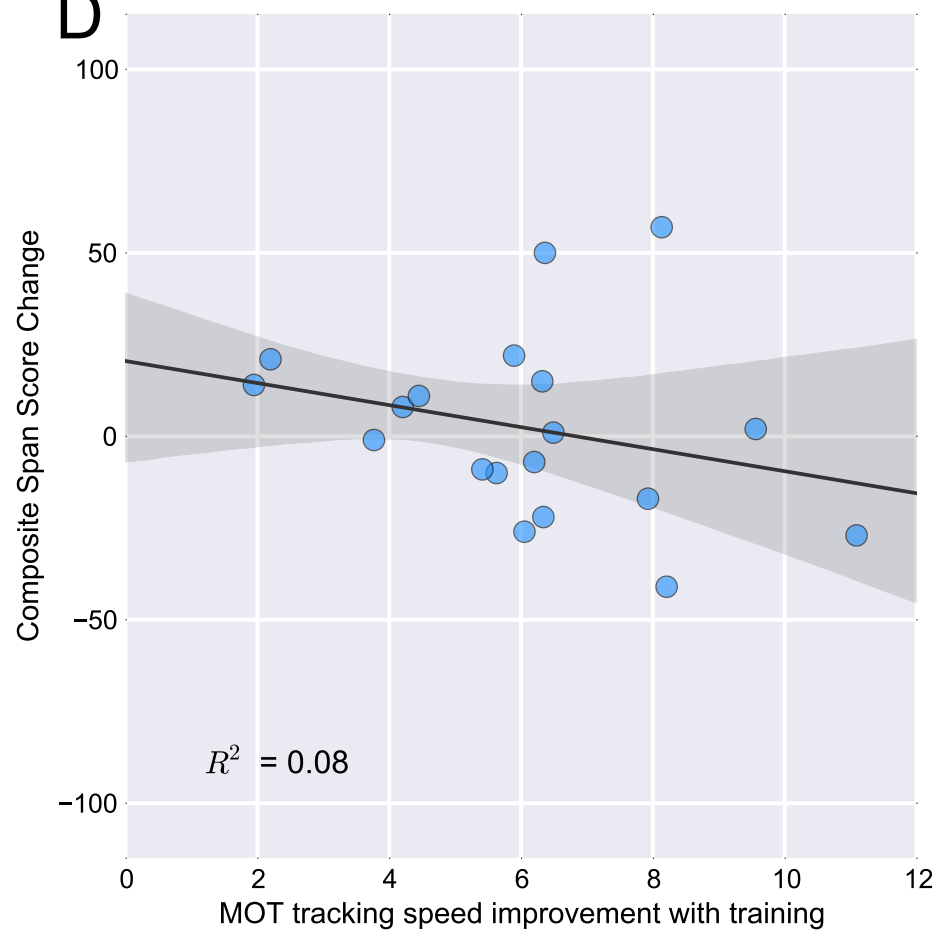

Supplement: Figure S2 — Relationships Between Training Gains and Transfer Measures. A) Correlation between improvement on the dual n-back task during training and the difference between pre- and post-training Ravens Advanced Progressive Matrices (RAPM) scores. B) Correlation between dual n-back improvement and change on the Composite Span Task scores. C) Correlation between improvement in Multiple Object Tracking (MOT) speed and RAPM change. D) Correlation between MOT gains and Composite Span Task score changes. All p’s >.05. Error bands are bootstrapped 95% confidence intervals for the regression. (PDF) [file pone.0063614.s002.pdf]
